# Real-World Comparative Effectiveness of Nivolumab versus Pembrolizumab in Patients with Unresectable Hepatocellular Carcinoma

**DOI:** 10.3390/pharmaceutics14112263

**Published:** 2022-10-23

**Authors:** Hsin-Yu Kuo, Meng-Zhi Han, Chih-Hsiang Liao, Yih-Jyh Lin, Chung-Teng Wang, Shang-Hung Chen, Ting-Tsung Chang, Po-Jun Chen, Sheng-Hsiang Lin, Chiung-Yu Chen, Chiao-Hsiung Chuang, I-Chin Wu, Juei-Seng Wu, Tzu-Chun Hong, Ming-Tsung Hsieh, Yang-Cheng Lee, Hung-Tsung Wu, Hong-Ming Tsai

**Affiliations:** 1 Department of Internal Medicine, National Cheng Kung University Hospital, College of Medicine, National Cheng Kung University, Tainan 70101, Taiwan; 2 Institute of Clinical Medicine, College of Medicine, National Cheng Kung University, Tainan 70101, Taiwan; 3 Department of Internal Medicine, An Nan Hospital, China Medical University, Tainan 70965, Taiwan; 4 Department of Environmental Resources Management, Chia Nan University of Pharmacy and Science, Tainan 71710, Taiwan; 5 Department of Surgery, National Cheng Kung University Hospital, College of Medicine, National Cheng Kung University, Tainan 70101, Taiwan; 6 Department of Oncology, National Cheng Kung University Hospital, College of Medicine, National Cheng Kung University, Tainan 70456, Taiwan; 7 National Institute of Cancer Research, National Health Research Institutes, Tainan 70456, Taiwan; 8 Department of Public Health, College of Medicine, National Cheng Kung University, Tainan 70101, Taiwan; 9 Biostatistics Consulting Center, National Cheng Kung University Hospital, College of Medicine, National Cheng Kung University, Tainan 70101, Taiwan; 10 Department of Internal Medicine, Tainan Municipal Hospital, Tainan 70103, Taiwan; 11 Department of Internal Medicine, School of Medicine, College of Medicine, National Cheng Kung University, Tainan 70101, Taiwan; 12 Department of Diagnostic Radiology, National Cheng Kung University Hospital, College of Medicine, National Cheng Kung University, Tainan 70101, Taiwan

**Keywords:** hepatocellular carcinoma, immune checkpoint inhibitors, immunotherapy, nivolumab, pembrolizumab, programmed cell death protein-1

## Abstract

Purpose: Immune checkpoint inhibitors are effective therapies for advanced hepatocellular carcinoma (HCC); however, comparisons of the clinical efficacy and safety profile for these drugs are still scarce. Thus, the aims of this study were to investigate the differences in efficacy and safety between nivolumab and pembrolizumab in unresectable HCC patients in a real-world setting. Patients and methods: A total of 115 patients who received treatment with nivolumab (n = 73) or pembrolizumab (n = 42) in combination with or without tyrosine kinase inhibitors was enrolled. Therapeutic response, survival outcomes, and safety profiles were compared among these groups. Multivariate analysis of survival response was performed using Cox proportional hazards regression. Results: Patients treated with pembrolizumab demonstrated a significantly higher objective response rate than those with nivolumab (38.1% vs. 15.1%; odds ratio 4.18, *p* = 0.005) regardless of the combination strategies. In addition, pembrolizumab performed a better overall survival (OS) than nivolumab, (34.9 vs. 9.5 months; hazard ratio (HR) = 0.39, *p* = 0.004). In subgroup analysis, pembrolizumab exhibited favorable OS than nivolumab for monotherapy (HR = 0.16, *p* = 0.001) or combination therapy (HR = 0.33, *p* = 0.006) as second-line or later-line (HR = 0.19, *p* = 0.001) therapy and those with (HR = 0.31, *p* = 0.006) or without (HR = 0.15, *p* = 0.004) well-compensated liver disease. The incidence of adverse events was comparable for both treatments. Conclusion: Both pembrolizumab and nivolumab had significant effects for HCC therapy, and pembrolizumab had a significant survival benefit as compared with nivolumab.

## 1. Introduction

With increasing incidence and mortality rates, hepatocellular carcinoma (HCC) is a global health challenge [1,2]. The prognosis of HCC differs markedly by tumor stage at diagnosis, and patients with an advanced tumor burden usually have a poor prognosis, with a median survival of only 1–2 years [1]. In order to combat HCC and increase the lifespan of the patients, systemic therapies with studies reporting a marked increase in overall survival (OS) and the quality of life of HCC patients were developed [3]. Among the available therapeutic options, immune checkpoint inhibitors (ICIs), which have been approved by the US Food and Drug Administration for the treatment of advanced unresectable HCC, represent a promising treatment choice [4,5].

Nivolumab and pembrolizumab, two monoclonal antibodies targeting programmed cell death protein 1 (PD-1), have shown encouraging efficacy and safety for the treatment of sorafenib-experienced HCC patients [6,7]. Studies have reported that an objective response rate (ORR) of 14–18% and a median OS of 12–15 months in advanced HCC patients treated with PD-1 inhibitors [6,7]. Although both nivolumab and pembrolizumab targeted to PD-1/PD-L1 signaling pathway [8], the binding site and affinity are different between these two inhibitors [9,10]. In addition, studies comparing the differences between anti-PD-1 regimens for HCC is scarce, and it remains crucial to identify any differences in both efficacies and their toxicity profiles for clinical use [11].

In this study, we conducted a head-to-head comparison to determine the efficacy and safety of anti-PD-1-based immunotherapies in a real-world cohort of patients with unresectable HCC and tried to provide suggestions to clinicians to select optimal inhibitors for patients.

## 2. Material and Methods

### 2.1. Study Subjects

This study was approved by the Institutional Review Board of National Cheng Kung University Hospital, Tainan, Taiwan (B-ER-110-300) and was performed according to the ethical principles for medical research of the World Medical Association Declaration of Helsinki.

A total of 211 participants with unresectable HCC were treated with ICIs at National Cheng Kung University Hospital (Tainan, Taiwan) from November, 2016 to December, 2020. ICIs were prescribed to participants with advanced HCC who had no history of systemic therapy or had progression after previous systemic regimens and to those with intermediate-stage HCC who experienced ineffective trans-arterial chemoembolization. This study included only participants with unresectable HCC, who were subsequently assessed using radiological imaging for tumor response [12,13].

**Figure 1:**
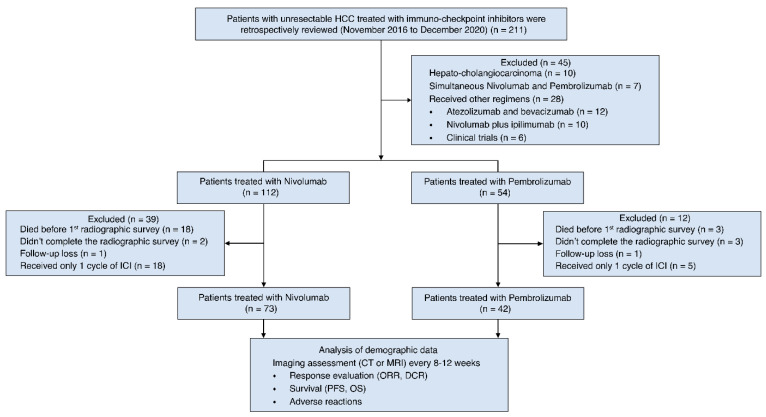
Flow diagram of patient selection. HCC, hepatocellular carcinoma; ICI, immuno-checkpoint inhibitors; CT, computed tomography; ORR, objective response rate; DCR, disease control rate; PFS, progression-free survival; OS, overall survival.

Among these 211 participants, 45 were excluded because of the proven pathology of hepato-cholangiocarcinoma (n = 10), simultaneous administration of nivolumab and pembrolizumab (n = 7), and administration of regimens other than nivolumab or pembrolizumab (n = 28). Of the remaining 166 patients, 51 were excluded because of death before the first assessment with radiological imaging (n = 21), incomplete radiologic evaluation (n = 5), loss to follow-up (n = 2), or receiving only one cycle of nivolumab or pembrolizumab (n = 23). The remaining 115 patients, comprising those treated with nivolumab (n = 73) and pembrolizumab (n = 42), met the study criteria and were enrolled in this retrospective analysis (Figure 1). The follow-up cutoff date was set as April, 2021. HCC diagnosis was based on tissue histology or typical radiographic findings [14]. Fine-needle cytology or biopsy was used for histological confirmation in cases of diagnostic uncertainty. Individuals not receiving the fine-needle cytology or biopsy were diagnosed with HCC by the hepatologist and clinician based on image studies and clinical presentations. 

### 2.2. Treatment and Response Evaluation

In this study, participants received a standard dose of 3 mg/kg of intravenous nivolumab biweekly or a fixed dose of 100 mg of intravenous pembrolizumab every 3 weeks. Adverse events were assessed according to the National Cancer Institute Common Terminology Criteria for Adverse Events (NCI CTCAE; version 5.0). The tumor response was evaluated on the basis of the Response Evaluation Criteria in Solid Tumors (RECIST) version 1.1 and the modified RECIST (mRECIST) based on serial contrast-enhanced computed tomography (CT) or magnetic resonance imaging (MRI) [15,16].

The ORR was defined as the percentage of patients with complete remission (CR) or partial response (PR) as the best overall response, and the disease control rate (DCR), the percentage of patients with at least CR, PR, or stable disease, as the best overall response. OS was calculated from the start of the PD-1 inhibitor treatment until the death of patients. Progression-free survival (PFS) was calculated from the start of treatment until tumor progression or death from any cause, whichever came first.

### 2.3. Statistical Analysis

The clinical characteristics of the two groups were compared using chi-square tests for categorical variables and unpaired Student’s *t* test or Wilcoxon rank-sum test for continuous variables. PFS and OS were compared between participants treated with nivolumab and pembrolizumab, respectively, using Kaplan–Meier curves and log-rank analysis. Logistic regression analysis was performed for the assessment of odds ratio (OR) for objective response, and Cox regression analysis was adopted to determine the hazard ratio (HR) for survival outcomes.

Multivariate Cox and logistic regression models were performed using propensity score, combination therapy (with or without tyrosine kinase inhibitors, including sorafenib, regorafenib, or lenvatinib), and the covariate of *p* < 0.1 recognized by stepwise regression. The covariates included age, sex, performance status, Child–Pugh class, Barcelona Clinic Liver Cancer classification, Cancer of the Liver Italian Program score, prior treatment, vascular invasion, combination with or without tyrosine kinase inhibitors (TKIs), and systemic line of immunotherapy. For the propensity score model, we fit a logistic regression model to estimate the probability of treatment for each participant, and we used the predicted probability as a weight in the multivariate regression model. The logistic regression model for the treatment was adjusted for the same covariates as above. A *p* value of <0.05 was considered statistically significant. All analyses were conducted using the SAS statistical package (v.9.4 for Windows; SAS Institute, Cary, NC, USA).

## 3. Results

### 3.1. Baseline Characteristics of the Study Subjects

In this study, the remaining 115 patients, comprising those treated with nivolumab (n = 73) and pembrolizumab (n = 42), we found that the median treatment time was 2.4 months for nivolumab (interquartile range (IQR), 1.8–6.1), and 3.9 months for pembrolizumab (IQR, 2.2–8.8). In addition, the median number of nivolumab and pembrolizumab administrations were 6.0 (range, 2–54 administrations) and 6.0 (range, 2–55 administrations), respectively. Median cumulative doses of nivolumab and pembrolizumab were 940 mg (range, 200–8680 mg) and 600 mg (range, 200–5550 mg), respectively.

Among the enrolled patients in the analyses, 42 (57.5%) patients treated with nivolumab and 24 (57.1%) receiving pembrolizumab were histologically confirmed hepatocellular carcinoma. The remaining 31 (42.4%) patients in the nivolumab group and 18 (42.8%) patients in the pembrolizumab group had a diagnosis of HCC based on imaging and clinical features.

As shown in Table 1, the baseline characteristics were not significantly different between patients treated with nivolumab and pembrolizumab. On enrollment, 55 patients (75.3%) treated with nivolumab and 32 (76.2%) receiving pembrolizumab were diagnosed as Child–Pugh class A (*p* = 1.000). Additionally, combination therapy of PD-1 inhibitor and a TKI was administered to 39 (53.4%) patients in the nivolumab group and 30 (71.4%) patients in the pembrolizumab group (*p* = 0.089). Eighteen (24.7%) and 14 (33.3%) patients received PD-1 inhibitors as first-line systemic therapy with nivolumab and pembrolizumab, respectively (*p* = 0.261).

### 3.2. Tumor Response

The overall ORR of patients treated with nivolumab and pembrolizumab in combination with or without TKIs were 17.8% and 40.5% (*p* = 0.015), respectively, based on mRECIST, and 15.1% and 38.1% (*p* = 0.010), respectively, based on RECIST. With regard to ICIs alone, the ORR was 14.7% for patients treated with nivolumab and 41.7% for those treated with pembrolizumab according to RECIST. Table 2 shows the unadjusted and adjusted OR for achieving an objective response in the nivolumab and pembrolizumab groups. The pembrolizumab group was more likely to achieve an objective response compared with the nivolumab group (OR = 3.61, *p* = 0.008). Multivariate analysis, including the adjustment of additional confounders in terms of using combination therapies with TKI regimens, revealed that pembrolizumab more significantly benefited the objective response compared to nivolumab (OR = 4.18, *p* = 0.005) (Model 2 in Table 2).

At the data cutoff date, five (6.8%) and five (11.9%) patients were still being treated with nivolumab and pembrolizumab, respectively. Immunotherapy was discontinued mainly because of disease progression (nivolumab and pembrolizumab, n = 42 [57.5%] and n = 14 [33.3%]) and change in treatment plan (nivolumab and pembrolizumab, n = 14 [19.2%] and n = 15 [35.7%]). After the discontinuation of immunotherapy, 39 (53.4%) in the nivolumab group and 28 (66.7%) in the pembrolizumab group received an alternative treatment. Two (2.7%) and four (9.5%) patients treated with nivolumab and pembrolizumab, respectively, subsequently underwent curative resection for the primary hepatic tumor. 

### 3.3. Survival

The median follow-up duration for the whole cohort was 8.9 (IQR, 5.0–17.0) months. The median PFS for the whole cohort was 3.5 (95% confidence interval [CI] 2.5–6.0) months, and the median OS was 11.8 (95% CI 8.9–15.4) months.

**Figure 2:**
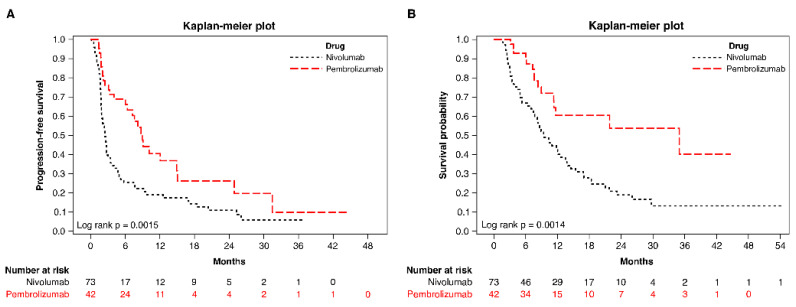
Progression-free survival (**A**) and overall survival (**B**) of patients treated with nivolumab or pembrolizumab.

For patients administered with ICIs combined with or without TKIs, the median PFS for patients treated with nivolumab was 2.5 (95% CI 1.9–3.0) months, whereas the median PFS for those treated with pembrolizumab was 8.9 (95% CI 6.0–14.9) months. The median OS was 9.5 (95% CI, 7.5–12.6) months for nivolumab and 34.9 (95% CI, 11.3–not estimable) months for pembrolizumab (Figure 2). 

Regarding ICIs alone, the median OS was 8.2 (95% CI 3.7–12.0) months for nivolumab and 21.8 (95% CI 7.3–not estimable) months for pembrolizumab (*p* = 0.031). In the adjusted Cox models, the adjusted HR for those receiving pembrolizumab in contrast to nivolumab was 0.44 (95% CI 0.27–0.71; *p* = 0.001) for PFS and 0.23 (95% CI 0.12–0.44; *p* = 0.001) for OS, as presented in Table 3. After particularly adjusting the confounding factor of combinative TKI therapy, the multivariate analysis demonstrated that pembrolizumab had a significantly more prognostic advantage as compared to nivolumab (adjusted HR = 0.48, *p* = 0.004 for PFS and adjusted HR = 0.39, *p* = 0.004 for OS) (Model 2 in Table 3).

### 3.4. Subgroup Analyses of Survival between Nivolumab- and Pembrolizumab-Treated Patients

Subgroup analyses of response and survival were conducted for patients treated with nivolumab or pembrolizumab, stratified by systemic line of immunotherapy, Child–Pugh class, and combination status. The ORR was significantly higher in patients treated with pembrolizumab than in those treated with nivolumab among patients in the second- or later-line therapy subgroup (42.9% vs. 10.9%, *p* = 0.002) and patients with Child–Pugh A cirrhosis (43.8% vs. 16.4%, *p* = 0.011).

**Figure 3:**
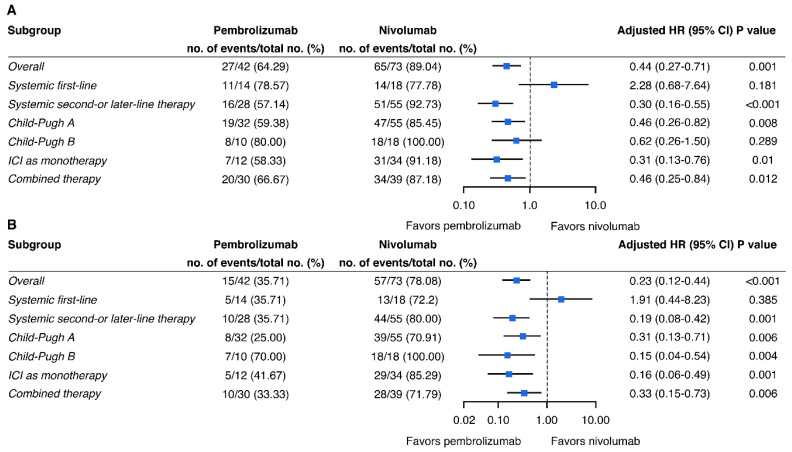
Hazard ratios (HR) of progression-free survival (**A**) and overall survival (**B**) for patients receiving pembrolizumab versus nivolumab. Squares represent subgroup-specific pooled HRs. Horizontal lines indicate a 95% confidence interval (CI).

Subgroup analyses with remarkable PFS were observed for pembrolizumab, when compared with nivolumab, in patients administered with PD-1 inhibitors as second- or further-line therapy (HR = 0.30, 95% CI 0.16–0.55), diagnosed with Child–Pugh A (HR = 0.46, 95% CI 0.26–0.82), and either ICI as a monotherapy (HR = 0.31, 95% CI 0.13–0.76) or in combination with TKI therapies (HR = 0.46, 95% CI 0.25–0.84) (Figure 3A). Except for the subgroup of patients receiving immunotherapy as a first-line therapy (HR = 1.91, 95% CI 0.44–8.23), a superior OS was observed for pembrolizumab over nivolumab, particularly in the subgroup of patients administered with PD-1 inhibitors as a second- or further-line therapy (HR = 0.19, 95% CI 0.08–0.42), between Child–Pugh class A (HR = 0.31, 95% CI 0.13–0.71) and class B (HR = 0.15, 95% CI 0.04–0.54) and either ICI as a monotherapy (HR = 0.16, 95% CI 0.06–0.49) or in combination with a TKI (HR = 0.33, 95% CI 0.15–0.73) (Figure 3B). Among the patients receiving PD-1 inhibitor monotherapy as their second-line treatment, the patients receiving pembrolizumab appeared to exhibit more favorable survival outcomes than those receiving nivolumab (adjusted HR = 0.24, *p* = 0.022 for PFS; adjusted HR = 0.07, *p* = 0.001 for OS).

### 3.5. Safety

The overall incidence of treatment-related adverse events for patients treated with nivolumab and pembrolizumab was 41.1% and 38.1%, respectively (*p* = 0.906). In both groups, the most common adverse events were skin rash, fatigue, and an increase in the aspartate aminotransferase level. No significant increase in the risk of grade 3/4 adverse events was observed between treatment with these two compounds.

## 4. Discussion

To the best of our knowledge, this study is the first report to compare treatment outcomes and safety profiles between different anti-PD-1 therapies in patients with unresectable HCC. Our results suggest that patients treated with pembrolizumab exhibit a longer OS compared with that of nivolumab, and each showed a comparable safety profile in unresectable HCC patients. The potential differences in efficacy across different anti-PD-1-based regimens may help clinicians to select the most appropriate drug for cancer therapy in clinical management.

As reported in nasopharyngeal carcinoma, these studies have shown a possible difference in efficacy and in the safety profiles across different anti-PD-1-based regimens, in which the ORR was higher for pembrolizumab than for nivolumab (26.3% vs. 19.0%) as a second- or later-line therapy, whereas the ORR with the first-line nivolumab reached as high as 40% [17]. The incidence rate of all-grade adverse events was lowest with nivolumab compared with pembrolizumab (54.2% vs. 74.1%). Unlike the previous studies, which consisted of the retrospective analysis in patients with melanoma or non-small cell lung cancer and showed no difference between the effectiveness of pembrolizumab and nivolumab [18,19]. There are currently no real-world studies that have compared the effectiveness between these two ICIs in HCC patients. Notably, our study has originality in illustrating the important implication that there could be heterogeneity in the treatment responses and clinical outcomes between these two PD-1 receptor inhibitors for HCC. Furthermore, a recent meta-analysis has reported that pembrolizumab contributed more to survival benefit as compared to nivolumab in advanced HCC patients (median OS 14.7 vs. 9.4 months) [20]. Their observations support our findings that pembrolizumab might deliver a superior treatment efficacy and survival outcomes over nivolumab, and supposed that the heterogeneity in the treatment responses might be specific to HCC patients. However, future prospective and large-scale studies are needed to validate the results of the current study. 

Pembrolizumab and nivolumab are both PD-1 receptor inhibitors that have demonstrated efficacy in several clinical trials [6,7,21]. Generally, the clinical practice of choosing suitable drugs is based on patients’ and clinicians’ preferences as well as adverse events. To date, there has not been a head-to-head randomized controlled trial on the efficacy differences between pembrolizumab and nivolumab to aid in therapeutic decision making. In this study, to illustrate the real-world setting of the unresectable HCC population, the efficacies of these two inhibitors were systemically analyzed and compared. Similar to a previous meta-analysis that reported that pembrolizumab contributes more to survival benefit compared with nivolumab in patients with advanced HCC [20], our study revealed that pembrolizumab had a superior ORR and favorable survival outcomes compared with nivolumab in various clinical settings. Furthermore, in agreement with previous studies dealing with various solid tumors, which have shown differences in the treatment efficacies between various PD-1-based therapies in clinical practice [17], our analyses indicated a possible difference in efficacy between these two drugs, which could provide important clues for treatment selection for clinicians in clinical practice. Therefore, these findings warrant further investigation, given their potential impact on drug selection for patients with HCC. Additionally, even with the nearly identical molecular structure between nivolumab and pembrolizumab, it has been shown that characteristics such as the binding site and binding affinity vary vastly between these inhibitors, which could impact antigen recognition and immunobiology in the tumor microenvironment [9,22]. Therefore, we believe that such variation might result in differences in the efficacy and safety profiles of different anti-PD-1-based strategies. However, due to the limited patient numbers, prospective studies detailing head-to-head comparisons are needed to verify the results of the current study.

Given the heterogeneity of HCC and with only some patients benefiting from immunotherapy, the current challenge is to identify the subset of HCC patients that these inhibiting agents may benefit the most, saving a substantial amount of health resources [1,11]. Uncertainty remains when using the available data to support treatment benefits and to guide the choice of immunotherapy in Child–Pugh class B patients [21]. Moreover, many combination strategies are being developed, and superior response rates have been reported when anti-PD-1 agents are combined with antiangiogenic targeted therapy [23]. However, limited biomarker data have been published to guide the treatment selection of ICIs or TKIs; a major unmet challenge is to identify the most beneficial ICI-antiangiogenic agent combination. The present study shows that treatment with pembrolizumab could result in better survival outcomes than nivolumab in unresectable HCC patients, irrespective of combination status or Child–Pugh class. The presence of additional differences among these agents may provide guidance in clinical management in patients with poor liver function reserve and for those treated with combination therapies. Future advancements in molecular profiling techniques and a better understanding of tumor biology and biomarkers could help to identify this subset of patients.

The ORR and prognosis in this study were superior to those reported by the KEYNOTE-240 trial (ORR 18.3%; OS 13.9 months) [24]. We believe these differences were primarily due to the higher proportion of patients having HCC due to viral hepatitis in this study compared with that in the KEYNOTE-240 trial (90.5% vs. 41.4%), and a lower proportion (52.4%) of patients receiving pembrolizumab as second-line systemic therapy in this study compared with that in the KEYNOTE-240 trial (second-line systemic therapy, 100%). Previously established studies exploring ICIs in patients with HCC have shown a trend toward a greater survival rate in patients with a viral hepatitis HCC etiology [24,25]. Furthermore, Kudo et al. have shown a favorable treatment response and survival outcomes with the use of pembrolizumab in Asian patients with HCC, which could be attributed to various complex factors, including geographical differences and a higher proportion of patients with a viral hepatitis HCC etiology [26]. Accordingly, the aforementioned aspects might support the efficacy differences between the current study and the KEYNOTE-240 trial.

Similar to published studies [6,27], the ORR of nivolumab alone in this study was 14.7%, despite 97.2% of the patients having underlying viral hepatitis. A previous study [28], which investigated the efficacy of nivolumab in the CheckMate 040 Asian cohort, showed that their treatment responses were consistent with those of the overall treatment population. Additionally, the responses and survival outcomes were similar across HCC etiologies [28], which suggests that the impact of nivolumab treatment in patients with HCC is minimally dependent on their hepatitis status. Hence, these observations deliver the important implication that there could be heterogeneity in the treatment response between these two PD-1 receptor inhibitors for HCC [29]. However, the small cohort size and the narrow scope of study population mainly enrollment of viral hepatitis B patients in the present study may not support our implication across other populations, particularly those lacking predominant hepatitis B virus infection. 

In this study, only patients who had subsequent imaging available for response evaluation were enrolled, which was consistent with the study design in previous studies reported by Lu et al. [12] and Lee et al. [13]. However, we agree that this exclusion of fatal patients before the first radiological imaging assessment might result in an immortal time bias. Hence, we performed survival analyses for patients, including 115 who had originally enrolled in the analyses, and 21 died before the first imaging. Among the total 136 patients, the median OS was 7.5 (95% CI 4.5–9.5) months for patients treated with nivolumab and 34.9 (95% CI 8.9–not estimable) months for those with pembrolizumab (*p* = 0.000). Their median PFS was 2.7 (95% CI 2.1–3.5) months for patients administered with nivolumab and 8.9 (95% CI 6.0–14.9) months for those administered with pembrolizumab (*p* = 0.003). In the adjusted Cox models, the HR for those receiving pembrolizumab in contrast to nivolumab was 0.47 (95% CI 0.28–0.78; *p* = 0.003) for PFS and 0.27 (95% CI 0.15–0.50; *p* = 0.001) for OS. In summary, these survival analyses results were consistent with the primary findings in our original design comprising 115 patients, and the exclusion process trivially affected the main conclusions regarding the survival efficacy between nivolumab and pembrolizumab.

Despite the significant findings in this study, there are several limitations to be addressed. First, the small cohort size and retrospective nature of the analysis may not better support this implication. However, the data presented in this study, together with previous reports, indicate a comprehensive understanding of the potential differences between these agents can result in clinical benefits, and our analysis may enable the selection and further development of optimal strategies with the best anticancer effects [22]. Additional studies with larger cohorts are needed. Second, the patients in our study were heterogeneous concerning the TKI regimen and prior therapies. However, after adjusting for combination therapies with TKIs, pembrolizumab remained an independently significant prognostic factor for treatment efficacy and survival. Further prospective investigations in a more homogenous and large-scale research are needed to confirm the comparative effectiveness of nivolumab and pembrolizumab for the treatment of unresectable HCC. Furthermore, the various treatment modalities post-progression or post-discontinuation might influence survival and treatment outcomes. Future head-to-head studies in a more homogeneous patient population are warranted for direct comparison.

## 5. Conclusions

In conclusion, pembrolizumab appears to exhibit favorable survival outcomes and a comparable safety profile in patients with unresectable HCC, compared with nivolumab. This understanding of the individual agents will assist in designing personalized therapy and could provide a valuable guide for future treatment strategies. However, because of the limited patient numbers in our analyses, further large cohort-based, long-term follow-up studies are required to determine the exact relationship between regional differences and treatment response.

## Figures and Tables

**Table 1:** Baseline characteristics of all patients.

Characteristic	Nivolumab (n = 73)	Pembrolizumab (n = 42)	*p*
	Number (%)	Number (%)	
**Gender**			
Female	55 (75.3)	34 (81.0)	0.645
Male	18 (24.7)	8 (19.1)	
**Age, years**—Mean ± SD	62.7 ± 11.4	63.0 ± 11.8	0.893
<55	15 (20.6)	12 (28.6)	0.454
≥55	58 (79.5)	30 (71.4)	
**ECOG**			
0	47 (64.4)	21 (50.0)	0.263
1	23 (31.5)	18 (42.9)	
2	3 (4.1)	2 (4.8)	
3	0 (0.0)	1 (2.4)	
**α-Fetoprotein, ng/mL**			
<400	44 (60.3)	23 (54.8)	0.703
≥400	29 (39.7)	19 (45.2)	
**Etiology of chronic liver disease**			
No liver disease	3 (4.1)	4 (9.5)	0.257
Liver disease present	70 (95.9)	38 (90.5)	
Chronic hepatitis B	51 (72.9)	30 (79.0)	0.642
Chronic hepatitis C	20 (28.6)	8 (21.1)	0.534
Alcoholic hepatitis	4 (5.7)	5 (13.2)	0.273
Nonalcoholic steatohepatitis	1 (1.4)	1 (2.6)	1.000
**Child–Pugh class**			
A	55 (75.3)	32 (76.2)	1.000
B	18 (24.7)	10 (23.8)	
**BCLC stage**			
B	12 (16.4)	4 (9.5)	0.452
C–D	61 (83.6)	38 (90.5)	
**CLIP**			
0–1	33 (45.2)	21 (50.0)	0.763
2–5	40 (54.8)	21 (50.0)	
**Distant metastases**			
No	33 (45.2)	15 (35.7)	0.425
Yes	40 (54.8)	27 (64.3)	
Lung	27 (67.5)	16 (59.3)	0.667
Bone	7 (17.5)	2 (7.4)	0.295
Brain	1 (2.5)	0 (0.0)	1.000
Lymph node	11 (27.5)	10 (37.0)	0.433
Other ^a^	18 (45.0)	7 (25.9)	0.185
**Prior treatment**			
No	10 (13.7)	5 (11.9)	1.000
Yes	63 (86.3)	37 (88.1)	
Surgical resection	33 (52.4)	16 (43.2)	0.499
TACE	43 (68.3)	20 (54.1)	0.228
RFA/PEI	25 (39.7)	10 (27.0)	0.287
HAIC	23 (36.5)	19 (51.4)	0.214
TKI	55 (87.3)	28 (75.7)	0.223
Systemic chemotherapy	2 (3.2)	0 (0.0)	0.529
Radioembolization	1 (1.6)	3 (8.1)	0.142
Radiotherapy	15 (23.8)	6 (16.2)	0.518
Clinical trial	2 (3.2)	2 (5.4)	0.625
Liver transplantation	1 (1.6)	0 (0.0)	1.000
**Macrovascular invasion**			
No	46 (63.0)	26 (61.9)	1.000
Yes	27 (37.0)	16 (38.1)	
**PD-1 inhibitors combined with TKI**			
No	34 (46.6)	12 (28.6)	0.089
Yes	39 (53.4)	30 (71.4)	
Sorafenib	22 (56.4)	6 (20.0)	0.003
Regorafenib	4 (10.3)	5 (16.7)	0.488
Lenvatinib	13 (33.3)	19 (63.3)	0.016
**PD-1 inhibitors as systemic therapy**			
First line	18 (24.7)	14 (33.3)	0.261
Second line	49 (67.1)	22 (52.4)	
Third line	5 (6.9)	6 (14.3)	
Fourth line	1 (1.4)	0 (0.0)	
**Median cycles received** (range)	6 (2–54)	6 (2–55)	0.835
**Median cumulative dose, mg** (range)	940 (200–8680)	600 (200–5550)	0.008

^a^ In nivolumab group: 9, 4, 4, and 1 patient with peritoneal metastases, adrenal involvement, retroperitoneal soft tissues, and diaphragm invasion, respectively; in pembrolizumab group: 4 and 3 patients with peritoneal metastases and adrenal involvement, respectively. ECOG, Eastern Cooperative Oncology Group performance status; CLIP, Cancer of the Liver Italian Program Scoring System; BCLC, Barcelona Clinic Liver Cancer; TACE, transcatheter arterial chemoembolization; RFA, radiofrequency ablation; PEI, percutaneous ethanol injection; HAIC, hepatic arterial infusion chemotherapy; PD-1, programmed cell death protein-1; TKI, tyrosine kinase inhibitor; SD, standard deviation.

**Table 2:** Adjusted odds ratio (OR) of achievement of objective response among the patients treated with nivolumab and pembrolizumab.

	Total (n = 115)		
Objective Response ^a^	Nivolumab (n = 73)	Pembrolizumab (n = 42)	*p*
n (%)	11 (15.07)	16 (38.10)	0.010
Unadjusted OR (95% CI)	1	3.47 (1.42–8.48)	0.006
Adjusted model 1 ^b^	1	3.61 (1.40–9.29)	0.008
Adjusted model 2 ^c^	1	4.18 (1.53–11.44)	0.005

^a^ Response was evaluated using the RECIST. ^b^ Adjustments were made including the predicted probability of propensity score and other covariates selected by stepwise logistic regression for *p* < 0.1. The covariates included age, gender, performance status, Child–Pugh class, Barcelona Clinic Liver Cancer (BCLC) classification, Cancer of the Liver Italian Program (CLIP) score, prior treatment, vascular invasion, combination with or without tyrosine kinase inhibitors (TKIs), and systemic line of immunotherapy. ^c^ Adjustments were made including the predicted probability of propensity score, categorical variable of TKI (none, sorafenib, regorafenib, and lenvatinib) and other covariates selected by stepwise logistic regression for *p* < 0.1. The covariates included age, gender, performance status, Child–Pugh class, BCLC classification, CLIP score, prior treatment, vascular invasion, and systemic line of immunotherapy. CI, confidence interval.

**Table 3:** Hazard ratio for progression-free survival (PFS) and overall survival (OS) for receiving pembrolizumab versus nivolumab.

	PFS	*p*	OS	*p*
Unadjusted Cox Model	0.49 (0.31–0.77)	0.002	0.41 (0.23–0.72)	0.002
Adjusted Cox model 1 ^a^	0.44 (0.27–0.71)	0.001	0.23 (0.12–0.44)	0.001
Adjusted Cox model 2 ^b^	0.48 (0.29–0.79)	0.004	0.39 (0.21–0.74)	0.004

^a^ Adjustments were made including the predicted probability of propensity score and other covariates selected by stepwise regression for *p* < 0.1. The covariates included age, gender, performance status, Child–Pugh class, Barcelona Clinic Liver Cancer (BCLC) classification, Cancer of the Liver Italian Program (CLIP) score, prior treatment, vascular invasion, combination with or without tyrosine kinase inhibitors (TKIs), and systemic line of immunotherapy. ^b^ Adjustments were made including the predicted probability of propensity score, categorical variable of TKI (none, sorafenib, regorafenib, and lenvatinib) and other covariates selected by stepwise regression for *p* < 0.1. The covariates included age, gender, performance status, Child–Pugh class, BCLC classification, CLIP score, prior treatment, vascular invasion, and systemic line of immunotherapy.

## Data Availability

Data are available on reasonable request to the corresponding author.
